# Wdr17 Regulates Cell Proliferation, Cell Cycle Progression and Apoptosis in Mouse Spermatocyte Cell Line

**DOI:** 10.3390/ani14101418

**Published:** 2024-05-09

**Authors:** Xin Zhao, Taili Jin, Xi Ji, Qiuyan Zhang, Xianyu Zhang, Zhenfang Wu, Zicong Li, Huaqiang Yang

**Affiliations:** National Engineering Research Center for Breeding Swine Industry, College of Animal Science, South China Agricultural University, Guangzhou 510642, China; 20211024011@stu.scau.edu.cn (X.Z.); jtl@stu.scau.edu.cn (T.J.); 20212024009@stu.scau.edu.cn (X.J.); qiuyan@stu.scau.edu.cn (Q.Z.); 2010020066@zqu.edu.cn (X.Z.); wzf@scau.edu.cn (Z.W.)

**Keywords:** GC-2spd(ts) cells, Wdr17, cell cycle, apoptosis, spermatocyte, spermatogenesis

## Abstract

**Simple Summary:**

Spermatogenesis is a highly efficient process that depends on the self-renewal and regulated differentiation of spermatogonial stem cells (SSCs) within the seminiferous tubules of the male mammalian testis. Male fertility requires the production of millions of gametes through the meiotic division of spermatocytes. We discovered that knocking out Etv5 in mice alters the expression of Wdr17, a gene crucial for spermatocyte development, by integrating bulk transcriptome sequencing data from the testes of Etv5 knockout mice with previously published single-cell transcriptome sequencing results. The levels of Wdr17 are strongly linked to cell survival and proliferation in mouse spermatocyte cell line GC-2spd(ts). Wdr17 could be a valuable gene target for investigating and controlling the processes of meiosis and spermatogenesis.

**Abstract:**

We identified Wdr17 as a highly expressed gene in pachytene spermatocytes by transcriptomic analysis of mouse testis. Germ cell-deficient infertile mouse models had significantly reduced Wdr17 expression. We performed gene interference and overexpression in the mouse spermatocyte cell line GC-2spd(ts) and investigated how Wdr17 affects spermatocyte growth and development. Our results showed that Wdr17 suppression significantly decreased cell growth rate and increased cell apoptosis in GC-2spd(ts) cells. Wdr17 suppression also arrested the cell cycle at the G1 phase. On the contrary, Wdr17 overexpression significantly promoted cell proliferation and inhibited cell apoptosis in GC-2spd(ts) cells. More cells were enriched at the S stage with a concomitant reduction of cells at the G1 stage. Wdr17 promotes mouse spermatocyte proliferation by advancing cell cycle progression and inhibiting cell apoptosis, indicating its potential role in regulating spermatogenesis in the mouse.

## 1. Introduction

Male fertility requires the production of millions of gametes through the meiotic division of spermatocytes. Each diploid spermatocyte produces four haploid spermatids by undergoing two meiotic divisions [1,2]. Meiotic division I begins in the leptotene stage of prophase, which occurs mainly in the basement membrane of the seminiferous tubules, after which the spermatocytes cross the blood–testis barrier into the lumen of the tubules, where they continue to develop into the zygotene, pachytene and diplotene stages. Each group of diploid primary spermatocytes differentiates into two haploid secondary spermatocytes, and the total number of chromosomes is reduced to half [3,4]. Meiotic division II does not involve DNA replication and occurs rapidly, with each haploid secondary spermatocyte differentiating into two haploid spermatids [3]. The meiotic division of testicular germ cells is a very important process in developmental biology. It ensures the genetic stability and diversity of the population, providing the basis for the reproduction and evolution of organisms.

Our previous studies have shown that ets variant 5 (Etv5) gene knockout in mice results in a blockage of spermatogenesis, leading to Sertoli cell- only syndrome [5]. Etv5 knockout mice showed progressive germ cell degeneration, with all germ cells depleted by 12 weeks of age [5,6]. The male infertility phenotype suggests that the Etv5 gene knockout disrupts spermatogenesis, probably by affecting germ stem cells. As these mice retain a complete spermatogenic cycle at the early stage of sexual maturation, they can be used as a model to study the molecular changes in the spermatogenic decay process. In addition to the stem cells, the spermatocytes and spermatids can also show significant changes in their gene expression profiles as germ cell depletion progresses, which can be used to identify the key genes that control the development of specific germ cells. 

By combining bulk transcriptome sequencing of testes from Etv5 knockout mice with published single-cell transcriptome sequencing results of mouse testicular cells [7,8], we found that Etv5 deficiency affects the expression pattern of Wdr17, whose expression is enriched in pachytene spermatocytes. We investigated how Etv5 regulates the expression of Wdr17, and how the changes in Wdr17 expression affect the proliferation and differentiation of mouse spermatocytes. We performed knockdown and overexpression assays of Wdr17 in the mouse spermatocyte cell line GC-2spd(ts) and found that knockdown of Wdr17 expression inhibited the proliferation of GC-2spd(ts) cells, whereas Wdr17 overexpression promoted the proliferation of GC-2spd(ts) cells. Wdr17 knockdown increased apoptosis of GC-2spd(ts) cells, whereas Wdr17 overexpression inhibited apoptosis. Furthermore, we found that changes in Wdr17 expression affected cell cycle progression in GC-2spd(ts) cells. This study reveals a novel regulator that controls mouse spermatocyte development, adding to our knowledge of spermatocyte biology. 

## 2. Materials and Methods

### 2.1. Bulk RNA-seq of Testes of Etv5 Knockout Mice

The global Etv5 gene knockout mice were prepared as per our previous publication [5]. The testes from 5-week-old wild-type and Etv5 knockout mice (4 mice for each genotype) were subjected to total RNA extraction. The poly(A) mRNA was enriched for the next-generation sequencing library preparations, as previously described [9]. RNA-seq was carried out using a 2 × 150 bp paired-end (PE) configuration in an Illumina HiSeq instrument. Low-complexity reads and technical sequences were filtered and processed by Cutadapt (v1.9.1) to be high-quality clean data. The clean data were aligned to a mouse reference genome (GRCm38) via the software Hisat2 (v2.0.1). HTSeq (v0.6.1) was used to estimate gene and isoform expression levels from the pair-end clean data, and the DESeq2 Bioconductor package was used for differential expression analysis.

### 2.2. Single-Cell RNA Sequencing and Cell-Type Assignment

We used the mouse adult testis scRNA-seq data from the public GEO database (GSE104556) to analyze the composition of mouse testicular cell types and identify their specific marker genes [7,8]. With the use of well-known molecular markers [10,11,12,13], we identified the major cell type populations in the mouse testis, which could be categorized into two main cell populations: germ cells and somatic cells. For the somatic cells, A genes were used to identify macrophages; Atca2 was used to identify peritubular and smooth muscle cells; Cyp11a1, Cyp17a1 and Star were used to identify Leydig cells; and Clu, Inha, Wt1 and Sox9 were used to identify Sertoli cells. For the germ cells, Dazl and Uchl1 were used to determine undifferentiated spermatogonia; Dmrt1 and Stra8 were used to determine differentiated spermatogonia; Syce1 was used to determine leptotene spermatocytes; Sycp1, Sycp3 and Tex101 were used to determine zygotene spermatocytes; Piwil1, Tmem30c, Mllt10, Rsph1 and Cdc42ep3 were used to determine pachytene spermatocytes; Aurka and Rassf1 were used to determine diplotene spermatocytes; Acrv1, Spaca1 and Lrriq1 were used to determine round spermatids; and Tnp1, Tnp2, Prm1, Prm2 and Spata3 were used collectively to determine elongating spermatid states. Subsequently, we used the “sc.tl.rank_genes_groups” function (a Wilcoxon rank-sum (Mann–Whitney U)) to compute a ranking for the highly differential genes in each cell type. We performed an integrative gene expression analysis by combining bulk and single-cell transcriptomics and obtained a list of differentially expressed genes (DEGs) in pachytene spermatocytes that were highly expressed in pachytene spermatocytes and associated with Etv5 mutant male infertility (Table 1).

### 2.3. Cell Culture and Transfection

Mouse spermatocyte-derived GC-2spd(ts) cells were obtained from the National Collection of Authenticated Cell Cultures (SCSP-5055). The cells were cultured in DMEM (Dulbecco’s Modified Eagle Medium) high-glucose medium (Gibco, Grand Island, NY, USA. Catalog no. 21068028) containing 10% (*v*/*v*) fetal bovine serum (FBS, Gibco. Catalog no. 10099-141), and 1% (*v*/*v*) penicillin-streptomycin (Gibco. Catalog no. 15140-122). The cell cultures were maintained at 37 °C in a humidified atmosphere containing 5% CO_2_. For cell transfection, the cells were plated in 24-well plates 1 day prior to transfection. The plasmid or siRNA was transfected using Lipofectamine 3000 reagent (Invitrogen, Carlsbad, CA, USA. Catalog no. L3000015).

### 2.4. EdU Incorporation Assay

The BeyoClick EdU Cell Proliferation Kit with Alexa Fluor 555 (Beyotime, Shanghai, China. Catalog no. C0075S) was used to determine the cell proliferation ability according to the previous methodology. Briefly, GC-2spd(ts) cells were incubated with 10 µM of EdU for 2 h in a 5% CO_2_ incubator at 37 °C. The cells were then fixed with 4% paraformaldehyde (PFA) for 15 min at room temperature and permeabilized with 0.1% Triton X-100 for 10 min at room temperature. The cells were then exposed to 100 µL of click reaction solution by incubation for 30 min at room temperature in the dark. The cell nuclei were stained with Hoechst 33342, and the images were captured using fluorescence microscopy (E800, Nikon, Tokyo, Japan). Cell images of six areas were taken randomly under the fluorescence microscope, and the proportion of EdU-positive cells was calculated using ImageJ (v1.51J8, National Institutes of Health, Bethesda, MD, USA). The proliferation rate was defined by the percentage of EdU-positive cells.

### 2.5. CCK-8 Assay

GC-2spd(ts) cells were seeded in a 96-well cell culture plate at a density of 2000 cells per well. After transfection with Wdr17 siRNA or overexpressing vector pcDNA3.1-Wdr17, the proliferation potential of the GC-2spd(ts) cells was assessed by a Cell Counting Kit-8 (CCK-8, Beyotime. Catalog no. C0037). In brief, 10 μL of CCK-8 solution was added to each well and incubated in a 37 °C, 5% CO_2_ incubator for 2 hr. The absorbance of each well was measured at 450 nm using a microplate reader assay (Synergy H1, BioTek, Winooski, VT, USA).

### 2.6. RNA Extraction and Quantitative PCR (qPCR)

Total RNA of the GC-2spd(ts) cells was isolated using a Total RNA Kit I (Omega Bio-Tek, Guangzhou, China. Catalog no. R6834) according to the manufacturer’s instructions. Total RNA of 1 μg was converted to cDNA using the PrimeScript RT reagent Kit with gDNA Eraser (Takara, Dalian, China. Catalog no. RR047B). The mRNA expression levels of specific genes were then measured by qPCR using Taq Pro Universal SYBR qPCR Master Mix (Vazyme, Nanjing, China. Catalog no. Q712-02). The specific primer sequences used for the qPCR assays are shown in Table 2, and β-actin was used as an internal reference gene. All the reactions were performed in triplicate. Relative gene expression levels were calculated using the 2^−ΔΔCT^ method. 

### 2.7. Mitochondrial Membrane Potential and Apoptosis Detection

Cell apoptosis was detected using the Mitochondrial Membrane Potential and Apoptosis Detection Kit (Beyotime. Catalog no. C1071S) in the GC-2spd(ts) cells after transfection with Wdr17 siRNA or overexpressing vector pcDNA3.1-Wdr17, according to the manufacturer’s instructions. The cells were stained in Annexin V-FITC, Mito-Tracker Red CMXRos and Hoechst 33342 staining solution to label apoptotic, viable and total cells, respectively. The cells were incubated for 30 min at room temperature in the dark. Fluorescence images were captured by fluorescence microscopy (E800, Nikon). The positive cells were quantified by ImageJ software.

### 2.8. Flow Cytometry

To examine the cell cycle and apoptosis in GC-2spd(ts) cells, flow cytometry (FCM) was performed using the Cell Cycle and Apoptosis Analysis Kit (Beyotime. Catalog no. C1052) or the Annexin V-FITC Kit (Beyotime. Catalog no. C1062S). The cells with siRNA or overexpressing plasmid transfection were fixed with 4% PFA in a PBS solution for 10 min, washed with PBS, and then stained with Annexin V-FITC and propidium iodide (PI) or PI only for 20 min at room temperature to analyze the cell apoptosis or cell cycle, respectively. Apoptotic cells were defined as Annexin V-positive and PI-negative cells. Cell cycle distribution was analyzed using ModFit LT 5.0 Software (Verity, Bedford, MA, USA).

### 2.9. Statistical Analysis

Statistical significance and standard deviation (SD) of the data obtained were determined by a two-tailed Student’s *t*-test using GraphPad Prism (v8.0.1, GraphPad Software, Boston, MA, USA). All the data are presented as the mean ± SD, and *p* < 0.05 was considered statistically significant.

## 3. Results

### 3.1. Transcriptomics Reveals Critical Role of Wdr17 in Mouse Spermatocyte Development

**Figure 1:**
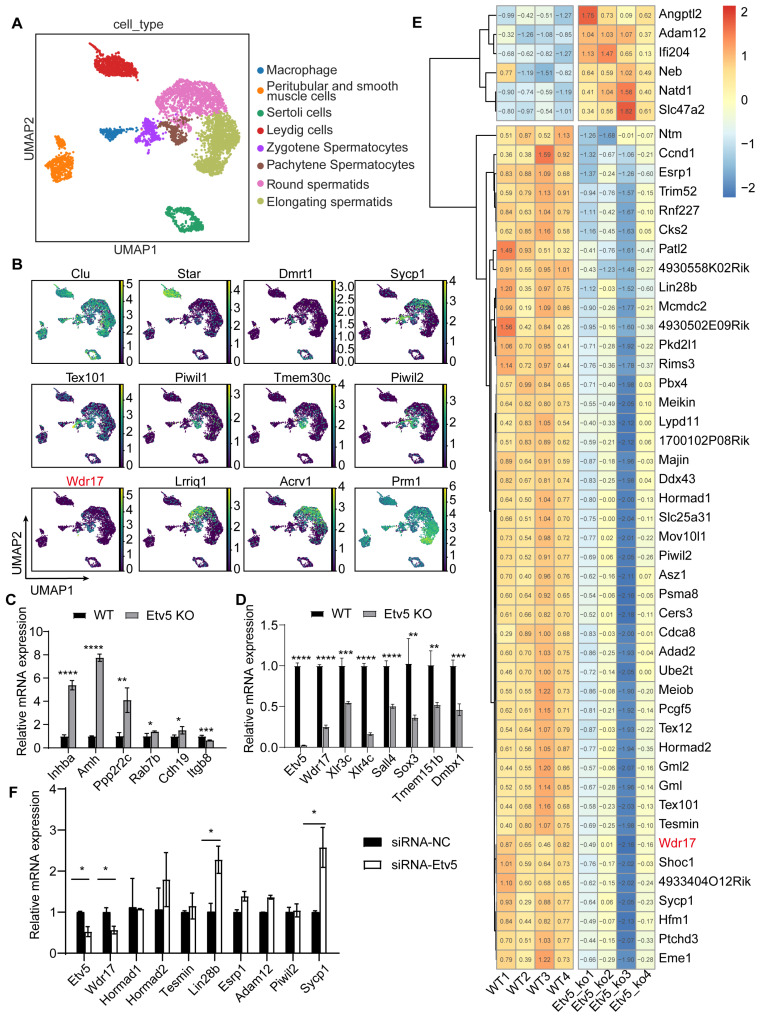
Wdr17 as a key regulator of mouse spermatocyte development identified by transcriptomic analysis. (**A**) UMAP plot of cells from adult mouse testis. (**B**) Gene expression patterns of representative markers for each cell type. (**C**) Relative expression levels of top upregulation genes in the Etv5 knockout testis validated by qPCR. (**D**) Relative expression levels of top downregulation genes in the Etv5 knockout testis validated by qPCR. (**E**) Heatmap of DEGs in pachytene spermatocytes determined by bulk RNA-seq and scRNA-seq. Numbers in the squares of heatmap are the normalized gene expression values. (**F**) Relative expression levels of selected pachytene spermatocyte DEGs in GC-2spd(ts) cells transfected with Etv5 siRNA for 48 h. Results are the mean ± SD from 3 testes for (**C**) and (**D**). Results are the mean ± SD of 3 wells of cultured cells. * *p* < 0.05, ** *p* < 0.01, *** *p* < 0.001, **** *p* < 0.0001; Student’s *t*-test (two-tailed).

We re-analyzed each major cell type from adult male mouse testis samples to provide an unbiased understanding of cellular heterogeneity using scRNA-seq data from the public GEO database (GSE104556) [7,8]. A set of previously described cell type-specific molecular markers was used to identify all the major testicular cell populations (see methods for details). We performed a nonlinear dimension reduction technique (uniform manifold approximation and projection, UMAP) and identified four major germ cell populations (zygotene spermatocytes, pachytene spermatocytes, round spermatids and elongating spermatids) and four somatic testicular cell populations (macrophages, peritubular and smooth muscle cells, Sertoli cells and Leydig cells) (Figure 1A). The typical gene markers used to identify these cell clusters are shown in Figure 1B. Previous research has shown that Etv5-null mice have lower body weights and a marked reduction in testis weight and size compared to wild-type controls, indicating germ cell lineage depletion and Sertoli cell-only syndrome [5,6]. We also used testicular bulk transcriptome sequencing to understand how Etv5 impacts the testis development. We confirmed the validity of the testicular bulk transcriptome data by qPCR validation of the top up- and downregulated genes (Figure 1C,D). Integrated analysis of the scRNA-seq and the bulk RNA-seq data revealed that Etv5 knockout affected the expression patterns of many spermatocyte-highly expressed genes, including Adam12, Hormad1, Hormad2, Tesmin, Wdr17, etc., indicating their important roles in mouse spermatocyte development (Figure 1E, Table 1). To validate the gene expression changes upon Etv5 deficiency, we used RNA interference for Etv5 knockdown and found a significant decrease in the Wdr17 mRNA level compared to other DEGs tested in GC-2spd(ts) cells (Figure 1F). Wdr17 was confirmed to mark pachytene spermatocytes in the scRNA-seq data (Figure 1B). In conclusion, transcriptomics and cell-based gene expression studies indicate that Wdr17 may be critical for mouse spermatocyte development.

### 3.2. Wdr17 Affects the Proliferation of GC-2spd(ts) Cells

**Figure 2:**
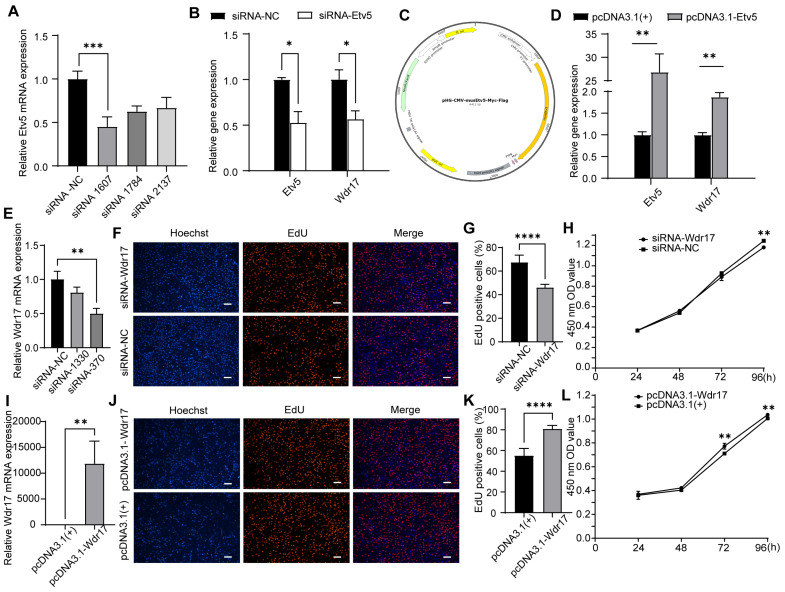
Wdr17 level affects GC-2spd(ts) cell proliferation. (**A**) Etv5 RNA interference effect in GC-2spd(ts) cells transfected with 3 different Etv5 siRNAs for 48 h.(**B**) Etv5 RNA interference decreased Wdr17 expression in GC-2spd(ts) cells. (**C**) Schematic diagram of the construction of Etv5 overexpressing vector pHG-CMV-musEtv5-Myc-Flag. (**D**) Etv5 overexpression increased Wdr17 expression in GC-2spd(ts) cells. (**E**) Wdr17 RNA interference effect in GC-2spd(ts) cells transfected with 2 different Wdr17 siRNAs for 48 h. (**F**) EdU staining showed that knockdown of Wdr17 inhibited the proliferation of GC-2spd(ts) cells. (**G**) Quantification of EdU-positive cells with Wdr17 interference by ImageJ. (**H**) CCK-8 assay showed that knockdown of Wdr17 inhibited the proliferation of GC-2spd(ts) cells. (**I**) Wdr17 mRNA level after overexpression by plasmid transfection in GC-2spd(ts) cells for 48 h. (**J**) EdU staining showed that overexpression of Wdr17 promoted the proliferation of GC-2spd(ts) cells. (**K**) Quantification of EdU-positive cells with Wdr17 overexpression by ImageJ. (**L**) CCK-8 showed that overexpression of Wdr17 promoted the proliferation of GC-2spd(ts) cells. Results are the mean ± SD of 3 replicates. * *p* < 0.05, ** *p* < 0.01, *** *p* < 0.001, **** *p* < 0.0001; Student’s *t*-test (two-tailed). Scale bars, 100 μm.

To investigate the effects of Etv5 on the gene expression pattern of Wdr17, we designed three siRNAs and verified their interference effects in GC-2spd(ts) cells (Figure 2A,B). We also used Etv5 overexpressing vector (pHG-CMV-musEtv5-Myc-Flag) to investigate the effects of Etv5 overexpression on the proliferation of GC-2spd(ts) cells (Figure 2C,D). The results showed that Wdr17 expression responds positively to Etv5 levels. Etv5 overexpression increased Wdr17 mRNA levels, whereas Etv5 knockdown suppressed Wdr17 mRNA levels in the GC-2spd(ts) cells (Figure 2B,D). To further determine the function of Wdr17 in GC-2spd(ts) cells, we designed two siRNAs for Wdr17 and verified their interference effects in the GC-2spd(ts) cells. The qPCR results showed that transfection of siRNA-3701 significantly inhibited the expression of Wdr17 mRNA in the GC-2spd(ts) cells (Figure 2E). The EdU and CCK-8 assays were used to examine the proliferation of GC-2spd(ts) cells after Wdr17 interference. Our results showed that siRNA-3701 significantly reduced GC-2spd(ts) cell proliferation (Figure 2F–H). We next constructed a Wdr17 overexpressing plasmid (pcDNA3.1-Wdr17). After plasmid transfection, qPCR assay results showed a significant increase in Wdr17 mRNA compared to blank vector transfection (Figure 2I). The EdU and CCK-8 assays revealed that Wdr17 overexpression significantly promoted the proliferation of GC-2spd(ts) cells (Figure 2J–L). In conclusion, Wdr17 can positively regulate the cell growth of mouse spermatocytes, in agreement with the phenotypic and transcriptomic data of Etv5 gene knockout mice.

### 3.3. Wdr17 Knockdown Promotes Apoptosis in GC-2spd(ts) Cells

**Figure 3:**
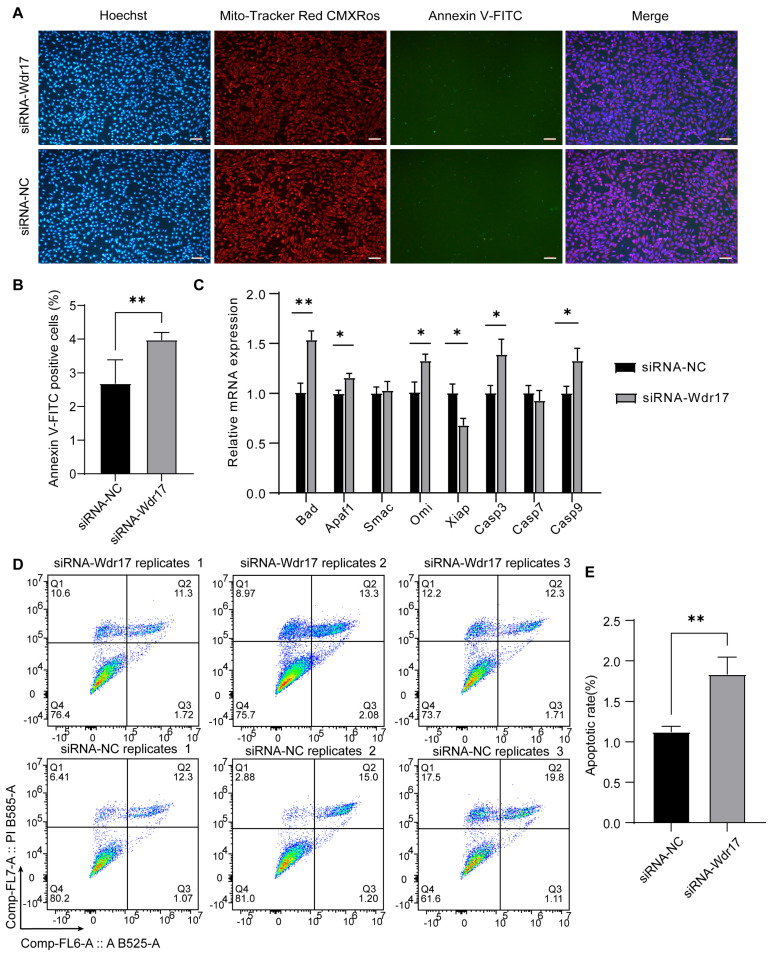
Wdr17 knockdown promotes apoptosis in GC-2spd(ts) cells. (**A**) Wdr17 knockdown promotes apoptosis in GC-2spd(ts) cells, labeled by Mito-Tracker Red CMXRos and Annexin V-FITC. (**B**) Quantification of the proportion of Annexin V-FITC-positive cells in GC-2spd(ts) cells with Wdr17 knockdown using ImageJ. (**C**) Wdr17 knockdown altered gene expression of apoptosis-related genes to promote apoptosis in GC-2spd(ts) cells. (**D**) FCM analysis of cell apoptosis by Annexin V-FITC and PI double staining of Wdr17-interfered GC-2spd(ts) cells. (**E**) Quantification of Annexin V^+^/PI^−^ apoptotic cells. Results are the mean ± SD of 3 wells of cultured cells. * *p* < 0.05, ** *p* < 0.01; Student’s *t*-test (two-tailed). Scale bars, 100 μm.

To investigate whether Wdr17 knockdown affects apoptosis in GC-2spd(ts) cells, we performed mitochondrial membrane potential and cell apoptosis detection in Wdr17 siRNA-transfected cells. Mito-Tracker Red CMXRos specifically labels biologically active mitochondria in cells, while Annexin V-FITC can label cells undergoing apoptosis. Following cell staining and data analysis, the Wdr17 knockdown group exhibited a greater number of apoptotic cells than the control group. This was evidenced by a higher proportion of Annexin V-FITC-positive cells in the knockdown group (Figure 3A,B). We performed qPCR analysis to detect the expression of apoptosis-related genes in the GC-2spd(ts) cells with Wdr17 interference. The results showed that Wdr17 knockdown significantly altered the mRNA expression of apoptosis-related genes in GC-2spd(ts) cells. Wdr17 knockdown significantly increased the mRNA levels of the pro-apoptotic factors Bad, Apaf1, Omi, Casp3 and Casp9, and decreased the mRNA expression of the anti-apoptotic factor Xiap, suggesting the activation of the apoptotic process by Wdr17 interference in GC-2spd(ts) cells (Figure 3C). We next confirmed the increased cell apoptosis by FCM analysis of Annexin V^+^/PI^−^ cells in Wdr17-interfered GC-2spd(ts) cells (Figure 3D,E).

### 3.4. Wdr17 Overexpression Inhibits Apoptosis in GC-2spd(ts) Cells

**Figure 4:**
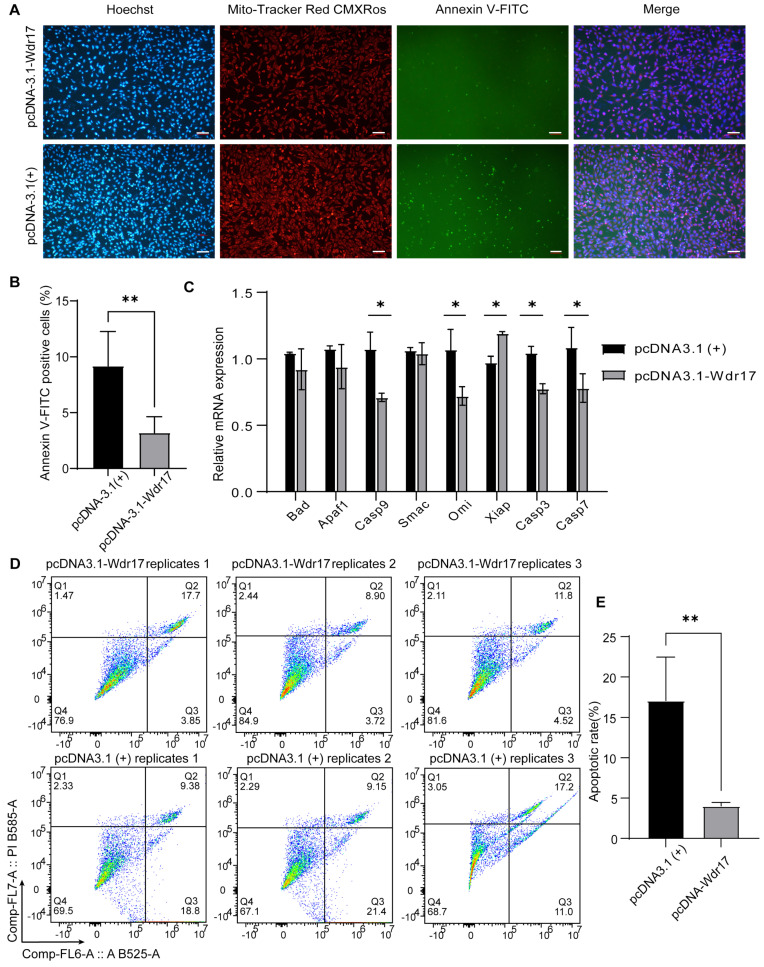
Wdr17 overexpression inhibits apoptosis in GC-2spd(ts) cells. (**A**) Wdr17 overexpression inhibits apoptosis in GC-2spd(ts) cells, measured by Mito-Tracker Red CMXRos and Annexin V-FITC staining. (**B**) Quantification of the proportion of Annexin V-FITC-positive cells in the GC-2spd(ts) cells transfected with pcDNA3.1-Wdr17 for 48 h. (**C**) Wdr17 overexpression inhibits cell apoptosis by affecting mRNA expression of apoptosis-related genes in GC-2spd(ts) cells. (**D**) FCM analysis of cell apoptosis by Annexin V-FITC and PI double staining of Wdr17-overexpressing GC-2spd(ts) cells. (**E**) Quantification of Annexin V^+^/PI^−^ apoptotic cells. Results are the mean ± SD of 3 wells of cultured cells. * *p* < 0.05, ** *p* < 0.01; Student’s *t*-test (two-tailed). Scale bars, 100 μm.

We next investigated the effect of Wdr17 overexpression on the level of apoptosis in GC-2spd(ts) cells. The cell staining results showed a significant decrease in apoptotic cells in the Wdr17 overexpression group compared to the blank vector-transfected control group (Figure 4A,B). A qPCR assay to detect the expression levels of apoptosis-related genes in GC-2spd(ts) cells transfected with pcDNA3.1-Wdr17 vector showed that Wdr17 overexpression significantly affected the mRNA expression of apoptosis-related genes in the GC-2spd(ts) cells. Wdr17 overexpression significantly inhibited the mRNA levels of pro-apoptotic factors Casp3, Casp7, Casp9 and Omi, and improved the mRNA expression of anti-apoptotic factor Xiap (Figure 4C). FCM analysis showed decreased Annexin V^+^/PI^−^ apoptotic cells upon Wdr17 overexpression (Figure 4D,E). Taken together, the results of Wdr17 overexpression and interference suggest that Wdr17 benefits mouse spermatocyte development by negatively regulating cell apoptosis.

### 3.5. Effects of Wdr17 on Cell Cycle Progression in GC-2spd(ts) Cells

**Figure 5:**
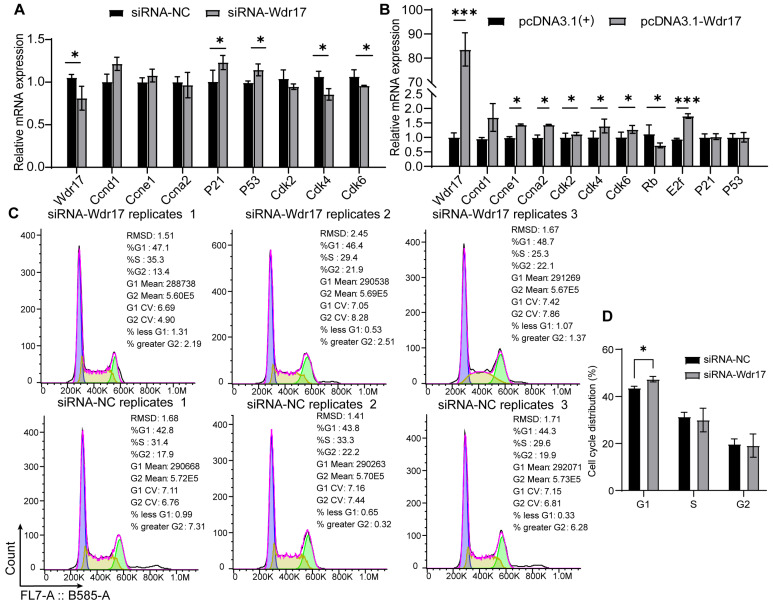

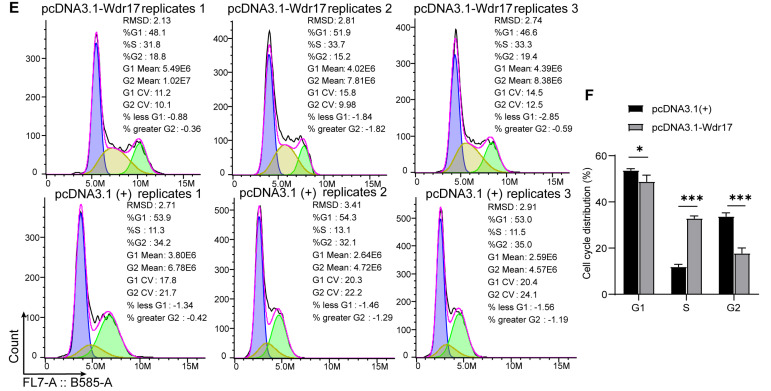
Effects of Wdr17 on cell cycle progression in GC-2spd(ts) cells. (**A**) Knockdown of Wdr17 alters expression of cell cycle-related genes in GC-2spd(ts) cells transfected with the Wdr17 siRNA. (**B**) Overexpression of Wdr17 alters the expression of cell cycle-related genes in GC-2spd(ts) cells transfected with the Wdr17 overexpressing vector. (**C**) FCM analysis of cell cycle distribution in Wdr17-knockdown GC-2spd(ts) cells. (**D**) Quantification of cell cycle distribution in Wdr17-knockdown GC-2spd(ts) cells. (**E**) FCM analysis of cell cycle distribution in Wdr17-overexpressing GC-2spd(ts) cells. (**F**) Quantification of cell cycle distribution in Wdr17- overexpressing GC-2spd(ts) cells. Results are the mean ± SD of 3 wells of cultured cells. * *p* < 0.05, *** *p* < 0.001; Student’s *t*-test (two-tailed).

Since cell proliferation is influenced by cell cycle status, we analyzed the influence of Wdr17 levels on cell cycle progression in GC-2spd(ts) cells. We found that Wdr17 knockdown resulted in a perturbation in the expression level of cell cycle-related genes. Wdr17 knockdown induced an increase in the expression of P53 and P21 and a decrease in the expression of CDK2, CDK4 and CDK6 (Figure 5A). Decrease in the expression of these CDKs could prevent the cell cycle transition from the G1 to the S phase, leading to cell cycle arrest in G1. In contrast, we found that Wdr17 overexpression promoted the expression of CDK2, CDK4, CDK6 and the corresponding cyclins, indicating promoted cell cycle progression. The decreased expression of cell cycle inhibitor Rb and the increased expression of transcription factor E2F also indicated accelerated cell cycle progression and cell proliferation upon Wdr7 overexpression (Figure 5B). FCM analysis showed that Wdr17 interference increased the G1 phase of the cell cycle (Figure 5C,D), whereas Wdr17 overexpression decreased the G1 phase and increased the S phase (Figure 5E,F), reflecting the role of Wdr17 in shortening the G1 phase to accelerate cell proliferation.

## 4. Discussion

Wdr17 is an understudied gene. Wdr17 has been reported to be expressed and functioned in the eyes [14,15,16]. Its biological function is not yet fully understood. Our work has shown that Wdr17 is highly expressed in pachytene spermatocytes in the mouse testis. Wdr17 plays a crucial role in maintaining the growth and function of mouse spermatocytes. Here, we used the sterile Etv5 knockout male mouse model to investigate DEGs that affect testis development [5]. To identify genes highly expressed in spermatocytes, we used previously published testicular scRNA-seq data [7,8]. Integrative analyses revealed that a number of genes were affected in spermatocytes in the sterile mouse model. This included significantly impaired Wdr17 mRNA expression, which was further validated when we performed Etv5 gene interference in a mouse spermatocyte cell line, GC-2spd(ts). 

Etv5-controlled Wdr17 may play a key role in spermatocyte development. We performed gene knockdown and overexpression assays and found that Wdr17 knockdown inhibited the proliferation of GC-2spd(ts) cells, whereas Wdr17 overexpression promoted the proliferation of GC-2spd(ts) cells. Wdr17 negatively regulated the apoptosis level of GC-2spd(ts) cells, suggesting the key role of Wdr17 in promoting spermatocyte proliferation. We also found that Wdr17 positively regulated the expression levels of cell cycle-related genes, including CDK2, CDK4 and CDK6. FCM analysis showed that Wdr17 overexpression decreased the G1 phase, whereas Wdr17 knockdown increased the G1 phase to induce a spermatocyte development arrest, indicating the positive role of Wdr17 in promoting cell cycle advancement in mouse spermatocytes. Despite the high expression of Wdr17 in mouse spermatocytes, Wdr17 may not be a specific marker gene for spermatocytes. In humans, WDR17 shows universal expression in various cells of the testis, with a relatively higher expression level in spermatocytes and spermatogonia (https://www.proteinatlas.org/search/WDR17 (accessed on 5 May 2024)). Therefore, Wdr17 may affect multiple cell types in the testis to regulate spermatogenesis, which needs further investigation.

Although our data show the importance of Wdr17 in regulating the growth level of GC-2spd(ts) cells, the true effect of Wdr17 on spermatocytes in vivo is uncertain and requires more study. The reported Wdr17-knockout mouse model did not show any significant phenotypic changes compared to wild-type mice. The Wdr17-knockout mice are fertile, and the proportions of wild-type, heterozygous knockout and homozygous knockout offspring are in accordance with Mendelian inheritance laws (https://www.mousephenotype.org/data/genes/MGI:1924662 (accessed on 5 May 2024)). These data suggest that Wdr17 deficiency may not severely affect spermatocyte development and spermatogenesis in vivo. Such a discrepancy could reflect the different mechanisms of action of Wdr17 between in vitro and in vivo contexts, or the existence of significantly different characteristics between GC-2spd(ts) and primary spermatocytes.

## 5. Conclusions

This study identifies Wdr17 as a critical regulator in the development of mouse spermatocytes; Wdr17 levels are closely related to cell survival and cell proliferation in mouse spermatocytes. Wdr17 may be a valuable gene target to study and manipulate the process of meiosis and spermatogenesis.

## Figures and Tables

**Table 1:** List of DEGs in pachytene spermatocytes from bulk RNA-seq and scRNA-seq. Genes are ranked by pachytene spermatocyte scores defined by scRNA-seq.

Gene Symbol	Transcriptomic Data of Etv5 Knockout Testis		Single-Cell Transcriptomic Data [8,9]			
	*p*vals_adj	log2FoldChange (knockout *vs.* wild-type)	Pachytene_Spermatocytes_*p*vals	Pachytene_Spermatocytes_Logfoldchanges	Pachytene_Spermatocytes_*p*vals_adj	Pachytene_Spermatocytes_Scores
Neb	0.035085187	1.115035706	7.94 × 10^−60^	4.2233295	5.80 × 10^−57^	16.313293
Piwil2	0.001805853	−1.210714668	2.27 × 10^−57^	4.5058355	1.40 × 10^−54^	15.964045
Tesmin	0.000433983	−1.340550875	6.41 × 10^−57^	4.729641	3.83 × 10^−54^	15.899256
Ntm	0.007700911	−1.033144584	4.68 × 10^−55^	3.2893615	2.50 × 10^−52^	15.628161
Hormad1	0.00123195	−1.394945387	2.88 × 10^−43^	3.8687394	8.59 × 10^−41^	13.791283
Tex101	0.000340654	−1.57479299	2.75 × 10^−37^	2.7778418	5.76 × 10^−35^	12.759866
Slc47a2	4.60 × 10^−5^	1.064626668	1.28 × 10^−31^	3.233085	2.05 × 10^−29^	11.69966
1700102P08Rik	0.004986544	−1.091706396	1.06 × 10^−29^	3.0629256	1.50 × 10^−27^	11.318685
Wdr17	0.013822108	−1.000954672	8.94 × 10^−29^	4.1073084	1.24 × 10^−26^	11.1301985
Hormad2	0.000508658	−1.45355141	2.03 × 10^−26^	4.056227	2.46 × 10^−24^	10.6358595
Pbx4	0.001542789	−1.033426469	6.58 × 10^−23^	3.5383756	6.33 × 10^−21^	9.854076
Adad2	0.000445121	−1.351408826	8.64 × 10^−23^	5.3795614	8.16 × 10^−21^	9.826668
Psma8	0.005144992	−1.152660427	8.63 × 10^−18^	3.5972211	5.87 × 10^−16^	8.590912
4933404O12Rik	0.001156883	−1.167389415	3.43 × 10^−17^	4.6093717	2.20 × 10^−15^	8.430986
Adam12	1.26 × 10^−9^	2.101374335	6.36 × 10^−17^	2.8372493	3.98 × 10^−15^	8.358305
Sycp1	0.002365455	−1.2813677	3.77 × 10^−15^	2.1614149	2.06 × 10^−13^	7.862474
Lypd11	0.005179153	−1.137202177	5.24 × 10^−15^	3.7817922	2.82 × 10^−13^	7.8209553
Ptchd3	0.000741707	−1.294829564	1.15 × 10^−13^	4.1466928	5.74 × 10^−12^	7.422454
Ifi204	0.000501088	1.391344816	3.01 × 10^−13^	3.1548893	1.46 × 10^−11^	7.293736
Mov10l1	0.000676886	−1.469099226	5.96 × 10^−12^	3.0391064	2.54 × 10^−10^	6.8805127
Esrp1	5.63 × 10^−8^	−2.353795628	1.48 × 10^−11^	3.4791603	6.07 × 10^−10^	6.749511
Lin28b	4.77 × 10^−5^	−1.589928027	1.48 × 10^−10^	3.0757754	5.43 × 10^−9^	6.4075217
4930558K02Rik	3.67 × 10^−5^	−1.121742116	8.09 × 10^−10^	2.089895	2.75 × 10^−8^	6.1432333
Patl2	0.002401623	−1.818866565	2.50 × 10^−9^	3.3682396	8.02 × 10^−8^	5.961757
Mcmdc2	0.00023271	−1.46087782	2.40 × 10^−8^	3.0477593	6.87 × 10^−7^	5.5803294
Gml	0.000163156	−1.650853404	3.19 × 10^−8^	2.3788683	9.05 × 10^−7^	5.5304885
Asz1	0.006742716	−1.180976405	4.03 × 10^−8^	3.0195432	1.13 × 10^−6^	5.4895577
Cers3	0.006782458	−1.103098215	4.26 × 10^−8^	3.4379182	1.19 × 10^−6^	5.479924
Cks2	0.000508101	−1.034688964	1.00 × 10^−7^	2.1928904	2.66 × 10^−6^	5.3263083
Natd1	6.71 × 10^−6^	1.07067814	5.05 × 10^−7^	2.3129487	1.21 × 10^−5^	5.024413
Shoc1	0.002652382	−1.264923666	3.73 × 10^−6^	2.813658	7.96 × 10^−5^	4.6260476
Ube2t	0.002919971	−1.200702292	4.96 × 10^−6^	2.5175054	0.000104762	4.566415
Rims3	0.000578389	−1.02391401	5.28 × 10^−6^	4.227404	0.000110944	4.5532537
Rnf227	3.63 × 10^−5^	−1.479084615	5.48 × 10^−6^	3.7630215	0.00011478	4.545429
Gml2	0.00281273	−1.445208949	7.47 × 10^−5^	2.2389789	0.001264598	3.9608386
Pkd2l1	0.001499767	−1.038933229	0.000105601	3.7833967	0.001722635	3.8773482
Ccnd1	0.000782232	−1.027401117	0.000191913	2.944979	0.00295234	3.7294312
Pcgf5	0.00025788	−1.40635133	0.00019446	2.1380026	0.002984545	3.726107
Meikin	0.004490066	−1.012027443	0.000229154	3.6511478	0.00345783	3.6844974
Cdca8	0.0060417	−1.039677017	0.000235647	2.5353625	0.003550373	3.677374
Majin	0.001037518	−1.444400641	0.000259084	3.5810795	0.003879766	3.6531093
Slc25a31	0.002702153	−1.216286706	0.000301156	2.2676058	0.004455657	3.6143038
Angptl2	0.001980206	1.00202547	0.000315776	2.965626	0.004647562	3.602002
Ddx43	0.002753675	−1.111080912	0.000390876	3.4711144	0.005626907	3.5461683
Meiob	0.0005354	−1.553742147	0.000578764	2.8036695	0.008004477	3.4413757
Trim52	1.01 × 10^−5^	−1.221516545	0.000843951	2.2543037	0.011140829	3.3379626
Hfm1	0.003331661	−1.183825782	0.000905049	3.0592759	0.011781762	3.3184922
Eme1	0.00421195	−1.007867675	0.00189948	3.7456686	0.022814588	3.105515
4930502E09Rik	0.034908872	−1.256370566	0.002200087	4.013551	0.025873651	3.0618024
Tex12	0.000777606	−1.504324304	0.002631263	3.172405	0.030188345	3.007823

**Table 2:** qPCR primers.

Genes	Primer Sequence (5′ to 3′)	mRNA Accession No.
Xiap	F: CGAGCTGGGTTTCTTTATACCG R: GCAATTTGGGGATATTCTCCTGT	NM_001301639.1
Smac	F: ATGACAGCGGTTGGCCTTT R: TCCTGTACCTGTGACTTCACC	NM_023232.3
Omi	F: TAGGACCCCGGATCTCTGG R: GACCCCAACCCCACAACAG	NM_019752.3
Apaf1	F: AGTAATGGGTCCTAAGCATGTTG R: GCGATTGGGAAAATCACGTAAAA	NM_001042558.1
Bad	F: AAGTCCGATCCCGGAATCC R: GCTCACTCGGCTCAAACTCT	NM_007522.3
Esrp1	F: CAGTTTAACCAGTCAGTGAGCAATG R: TCAGGCAGTAACACATTCTTCTTGG	NM_001290383.1
Adam12	F: AAGTGTGGAAATGGCTATGTGGAAG R: GGTAGCGTTACAGCAGCGATTC	NM_007400.3
Hormad2	F: AGGACGATGGCACTACTGAGATAG R: TTCGCTGACCTTCTTCTTCTTTCTG	NM_001417965.1
Hormad1	F: AAGTGGATGCTTGGATGCTATGATG R: TTGTCTGAGGATCTCCTGGATTGG	NM_001289532.1
Tesmin	F: GGTGAGGAAGCAGAGCAGGAG R: GGACTTGAACTCGATGTGGAGAATC	NM_001039657.2
Lin28b	F: GGCCTTGAGTCAATACGGGT R: ATCCTGCCGTCTCCACCTAT	NM_001031772.2
β-actin	F: GGCTGTATTCCCCTCCATCG R: CCAGTTGGTAACAATGCCATGT	NM_007393.5
Ccnd1	F: ATTTCCAACCCACCCTCCAT R: GGGGTCCTTGTTTAGCCAGAG	NM_001379248.1
Ccne1	F: TGTTACAGATGGCGCTTGCT R: GCCAGGACACAATGGTCAGA	NM_007633.2
Ccna2	F: GTCAACCCCGAAAAACTGGC R: TTAAGAGGAGCAACCCGTCG	NM_009828.3
Cdk2	F: CGGAGTGGTGTACAAAGCCA R: TCGGATGGCAGTACTGGGTA	NM_016756.4
Cdk4	F: CCTGCCGGTTGAGACCATTA R: TCAGGTCCCGGTGAACAATG	NM_001355005.1
Cdk6	F: TCCTGCTCCAGTCCAGCTAT R: CCACGTCTGAACTTCCACGA	NM_009873.3
P53	F: CCATGGCCCCTGTCATCTTT R: TGAGGGGAGGAGAGTACGTG	NM_001127233.1
P21	F: GCAAAGTGTGCCGTTGTCTC R: CGTCTCCGTGACGAAGTCAA	NM_001111099.2
Dmbx1	F: GTTCCCACGGAGAAGGCAAGG R: TCCGACAGGCTCAGTTGAAGTTC	NM_001025567.1
Tmem151b	F: GAGGAGGACGAGGACGAGGAG R: GCCGATGGACGATGAGGACAG	NM_001013749.3
Sox3	F: ACAACTCCGAGATCAGCAAGCG R: TCCTTCTTGAGCAGCGTCTTGG	NM_009237.3
Sall4	F: CCAGGACGACGCAGCAGAAG R: CCAACACAGAGAAGCCCAGAGAG	NM_175303.5
Cdh19	F: TGGCTATCATGCTCGCATCCTATAC R: CAGACAAGGCTCCAGGCTGAC	NM_001081386.2
Rab7b	F: TGGGACACAGGTGGTCAGGAG R: AATGCCAGGATACAGCCATCGG	NM_001311096.1
Itgb8	F: GGGAGTGTGAAGGTGGCAGATG R: AGTGCTGTGCTGAGGCTGATG	NM_177290.4
Ppp2r2c	F: GGACGACCTACGCATCAACCTC R: TGCTGCTGCTGTAGACGAAGAG	NM_001360003.1
Amh	F: ACTCGCTTGGTTCGTGCTCTG R: GGGTGACAGCAGCAGTAATAGGG	NM_007445.3
Inhba	F: CTCGCTCTCCTTCCACTCAACAG R: AGCCACACTCCTCCACAATCATG	NM_008380.2
Xlr3c	F: ATCGCTGAAGAGCTGAGACG R: GTGGAGGAGCAGCAGTCTTT	NM_011727.2
Rb	F: TAACCTTGAACCTGCTTGTCCTCTC R: GGCTGCTTGTGTCTCTGTATTTGC	NM_009029.3
E2f	F: GTGGCTGCTGACTCACTCCTG R: TCTCTAATGCCCTCACCCTCCTC	NM_001291105.1
Casp9	F: TGAAGAACGACCTGACTGCCAAG R: ATGAGAGAGGATGACCACCACAAAG	NM_001277932.1
Casp3	F: GACTGGAAAGCCGAAACTCTTCATC R: AGTCCCACTGTCTGTCTCAATGC	NM_001284409.1
Casp7	F: ACTCCACGGTTCCAGGTTATTACTC R: AGGTCCTTGCCATGCTCATTCAG	NM_007611.3
Wdr17	F: TCCAGGTGGCAGTGACAACTTG R: TCGTTAGTTCTTGGGCTTCTGAGG	NM_001372372.1
Piwil2	F: TAAAACTCACCCCTCTGGTGC R: ACAGGATCCATTGAGAGGCT	NM_001364321.1
Sycp1	F: TGAGGGGAAGCTCACGGTT R: CGAACAGTGTGAAGGGCTTTTG	NM_011516.2
Etv5	F: AAGAATCGGCCAGCCATGAA R: TCCGGGAAGGCCATAGAGAA	NM_001358428.1

## Data Availability

The bulk RNA-seq data are deposited at the NCBI and Sequence Read Archive under BioProject PRJNA1102843. The scRNA-seq data were obtained from the GEO database with the identifier GSE104556. The other data that support the findings of this study are available on request from the corresponding authors.
